# The Impact of Endometrial Thickness on the Day of Human Chorionic Gonadotrophin (hCG) Administration on Ongoing Pregnancy Rate in Patients with Different Ovarian Response

**DOI:** 10.1371/journal.pone.0145703

**Published:** 2015-12-30

**Authors:** Zhiqin Bu, Yingpu Sun

**Affiliations:** Reproductive Medical Center, First Affiliated Hospital of Zhengzhou University, Zhengzhou, China; Institute of Zoology, Chinese Academy of Sciences, CHINA

## Abstract

In order to explore the impact of endometrial thickness on hCG administration day on ongoing pregnancy rate (OPR) in IVF-ET cycles, we retrospectively analyzed data from 10,406 patients undergoing their first IVF cycles with standard gonadotropin releasing hormone analogue (GnRH-a) long protocol. Firstly, patients were divided into poor (≤ 5 oocytes), medium (6–14 oocytes), and high (≥ 15 oocytes) ovarian responders based on the number of oocytes retrieved. In each group, patients were sub-divided into three groups according to the endometrial thickness on the day of hCG administration: Group A, thin endometrial thickness (≤ 7 mm); Group B, medium endometrial thickness (8–13 mm); Group C, thick endometrial thickness (≥ 14 mm). (1) For poor responders, OPRs were significantly different in the three endometrial thickness groups (28.57%, 44.25%, and 51.34%; *P* = 0.008). The association between thin endometrial thickness and OPR was significant after controlling for age, number of embryos transferred by multivariate logistic regression analysis (adjusted OR: 0.408; 95% CI: 0.186–0.898; *P* = 0.026. Reference = thick endometrial thickness). (2) For medium responders, OPRs were 31.58%, 55.56%, and 63.01% (*P* = 0.000) in the three groups. Adjusted OR for thin endometrial thickness was 0.284 (95% CI: 0.182–0.444; *P* = 0.000). (3) For high responders, OPRs were also significantly different in the three groups (28.13%, 52.63%, and 63.18; *P* = 0.000). Adjusted OR for thin endometrial thickness was 0.233 (95% CI: 0.105–0.514; *P* = 0.000). For patients undergoing IVF with different ovarian response, a thin endometrium on the day of hCG administration adversely affects ongoing pregnancy rate.

## Introduction

Embryo quality and endometrial receptivity are seemed to be two main factors associated with successful *in vitro* fertilization and embryo transfer (IVF-ET) cycle [1]. Ultra-sonographic examination, which is easily performed and noninvasive, has been routinely used as an alternative method to assess endometrial receptivity. Parameters used to evaluate endometrial receptivity include endometrial thickness, endometrial pattern, and endometrial and sub-endometrial blood flow [2,3,4].

Although many studies have evaluated the relationship between endometrial thickness and IVF outcome, the results are still controversial. Some authors reported no association between endometrial thickness and pregnancy rate in patients undergoing IVF [5,6], while others demonstrated a higher pregnancy rate at certain endometrial thickness [7,8,9]. In most studies, including a recent high quality Meta analysis, it is reported that thin endometrium adversely affects pregnancy rate in IVF [4,10,11].

There are several reasons why the findings from these studies were inconclusive: (i) patient basic parameters, such as female age, number of oocytes retrieved, quality and number of embryos transferred were not well evaluated in most studies; (ii) sample sizes in these retrospective studies were relatively limited; (iii) endometrial thickness was not measured in the same day during IVF (human chorionic gonadotropin administration day, oocyte retrieval day, embryo transfer day, etc.). Thus, in order to get a more accurate relationship between endometrial thickness and IVF outcome, we only included patients transferred with high quality cleavage stage embryo in standard GnRH-a long protocol, and divided them into three groups according to ovarian response, trying to control the impact of other pregnancy rate associated factors.

## Materials and Methods

### Patients

This work has been approved by the Institutional Review Board (IRB) of First Affiliated Hospital of Zhengzhou University in March 2015. For patients undergoing IVF treatment in our center, all of them have agreed to allow us to use their medical record data for research. A written informed consent was obtained from all patients before IVF treatment. In this study, patient records were anonymized and de-identified prior to data collection from our electronic database.

In total, 10,046 patients undergoing their first IVF/ICSI cycles treated with a standard GnRH-a long protocol between August 2009 and January 2015 at Reproductive Medical Center, First Affiliated Hospital of Zhengzhou University were included into this study. Only patients transferred with high quality embryos were included into this study. Exclusion criteria included: the presence of a known endometrial polyp or uterine anomaly; patients with uterine fibroid, Adenomyoma, or hydrosalpinx; oocyte donation cycles; pre-implantation genetic diagnosis (PGD) cycles.

### Treatment protocol and cleavage stage embryo scoring

All patients were treated with a standard GnRH-a long protocol. Details of this protocol were described previously [12].

Cleavage stage embryos were divided into 4 grades in our center [13]. Embryos scored with Grade I or Grade II were considered to be high quality embryo.

To assess IVF outcome, serum human chorionic gonadotropin (hCG) was measured 14 days and 18 days after embryo transfer. Clinical pregnancy was confirmed by ultrasound observation 3 weeks after positive hCG test. The luteal phase was supported with 60 mg IM of progesterone in oil, starting on the day of oocyte retrieval until 12 weeks’ gestation if pregnancy was achieved. An ongoing pregnancy was defined as a pregnancy with a positive heartbeat by ultrasound after 12 weeks of gestation.

### Statistical analysis

To explore the relationship between endometrial thickness and ongoing pregnancy rate (OPR) in all the 10,046 cycles, patients were divided into 5 distinct groups according to endometrial thickness on the day of embryo transfer: ≤7mm, 8-10mm, 10-12mm, 12-14mm, and ≥14mm. OPR was calculated for each endometrial thickness interval.

Then, the 10,046 cycles were firstly divided into poor (≤ 5 oocytes), medium (6–14 oocytes), and high (≥ 15 oocytes) ovarian responder groups based on number of oocytes retrieved. Patients in the three groups mentioned above were then sub-divided into three endometrial thickness groups (≤7mm, 8-13mm, ≥14mm).

ANOVA and chi-square test were used to evaluate differences in basic parameters and OPR between the three endometrial thicknesses groups separately for patients with different ovarian response. Multivariate logistic regression analysis was used to explore the association between endometrial thickness and OPR after controlling for age, and number of embryos transferred.

Statistical analysis was performed with SPSS (Statistical Package for Social Science, SPSS Inc, Chicago, IL, USA) version 17.0. *P*<0.05 was considered statistically significant.

## Results

A total of 10,046 cycles (poor responder: 1,399 cycles; medium responder: 6,501 cycles; high responder: 2,146 cycles) were included into this study. The incidences of thin endometrial thickness in these three groups were: 2.50% (35/1,399), 1.46% (95/6,501), and 1.49% (32/2,146), respectively.

**Fig 1:**
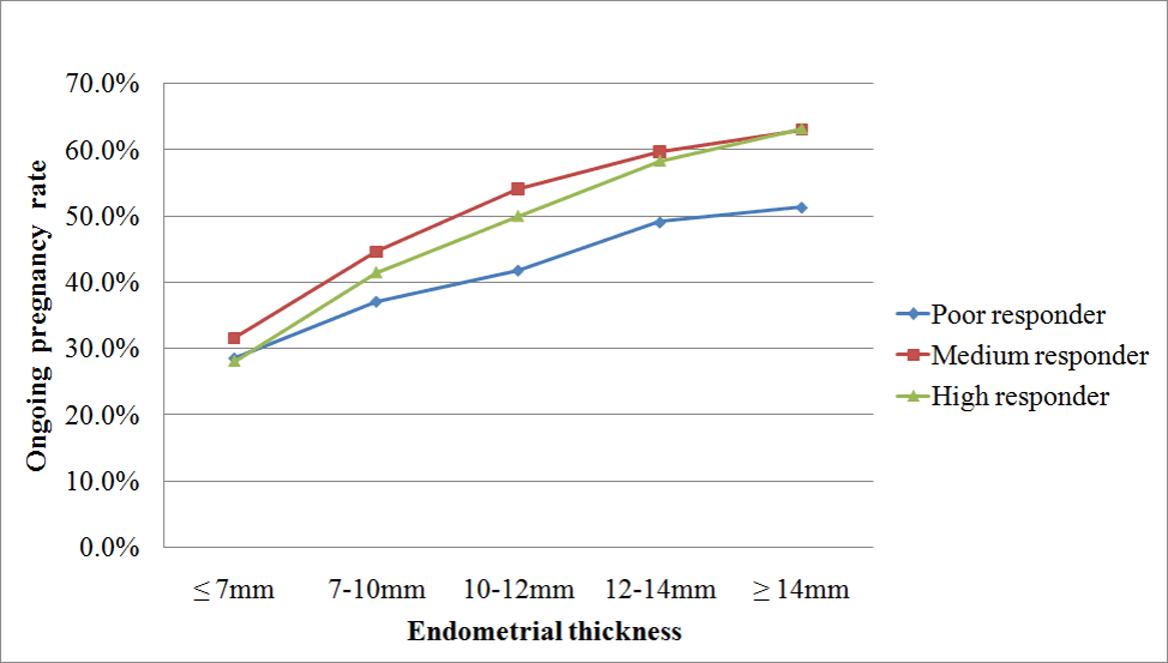
The relationship between endometrial thickness on the day of hCG administration and ongoing pregnancy in patient with different ovarian response.

Fig 1 showed the overall association between endometrial thickness on hCG administration day and OPR in 10,046 cycles. There was an elevation in OPR with progressively thicker of endometrial thickness in all the three groups. OPR seemed to be positively associated with endometrial thickness on hCG administration day.

For poor ovarian responders (n = 1,399), patients in the three different endometrial thickness groups had similar age, BMI, infertility duration, number of oocytes retrieved, and number of embryos transferred. However, endometrial thickness (6.71mm, 11.09mm, and 15.23mm; *P* = 0.000) and OPR (28.57%, 44.25%, and 51.34%; *P* = 0.008) were significantly lower in patients with thin endometrial thickness (Table 1). In addition, multivariate logistic regression analysis showed that endometrial thickness was still significantly associated with OPR after adjusting age and number of embryos transferred (Table 2).

**Table 1:** Basic characteristics and ongoing pregnancy rate for patients with poor ovarian response.

	Thin	Medium	Thick	*P*
	≤hick	8–13 mm	≥ 14mm	
	Group A	Group B	Group C	
No. of patients	35	992	372	
Age (y)	34.91 ± 5.30	33.69 ± 5.67	33.57 ± 5.49	0.399
BMI (Kg/m^2^)	23.03 ± 3.54	22.75 ± 3.22	22.69 ± 3.23	0.833
Duration of infertility (y)	4.44 ± 3.39	5.49 ± 4.33	5.42 ± 3.94	0.380
No. of oocytes retrieved	4.00 ± 1.09	3.96 ± 1.02	4.06 ± 0.97	0.277
No. of embryos transferred	2.00 ± .042	2.04 ± 0.34	2.02 ± 0.33	0.338
Endometrial thickness on hCG day	6.71 ± 0.61^+^ ^&^	11.09 ± 1.50^+^ ^Δ^	15.23 ± 1.40^&^ ^Δ^	0.000
Ongoing pregnancy rate (%)	28.57 (10/35)^&^	44.25(439/992)^Δ^	51.34 (191/372)^&^ ^Δ^	0.008

Note: data were mean ± standard deviation unless otherwise noted; BMI = body mass index; hCG = human chorionic gonadotrophin

^+^ Group A versus Group B; *P*<0.05

^&^ Group A versus Group C; *P*<0.05

^Δ^Group B versus Group C; *P*<0.05

**Table 2:** The impact of endometrial thickness on ongoing pregnancy rate by multivariate logistic regression analysis.

	Poor ovarian responders	Medium ovarian responders	High ovarian responders
	(*n* = 1,399)	(*n* = 6,501)	(*n* = 2,146)
	aOR (95% CI)	aOR (95% CI)	aOR (95% CI)
Age (y)	0.899 (0.881–0.919) **	0.938 (0.928–0.948) **	0.952 (0.932–0.972)**
No. of embryos transferred	2.126 (1.510–2.992) **	1.124 (0.932–1.355)	0.853 (0.566–1.286)
Endometrial thickness on hCG day			
Thick (≥ 14 mm)	1.000 (Reference)	1.000 (Reference)	1.000 (Reference)
Medium (8–13 mm)	0.728 (0.567–0.934) *	0.734 (0.656–0.822) **	0.651 (0.536–0.791)**
Thin (≤ 7mm)	0.408 (0.186–0.898) *	0.284 (0.182–0.444) **	0.233 (0.105–0.514)**

Note: hCG = human chorionic gonadotrophin; aOR = adjusted odds ratio; CI = confidence interval;

*P<0.05;

**P<0.01

For medium ovarian responders, even patients with thin endometrial thickness were a little older than patients in the other two groups (31.82, 30.64, and 30.50;*P* = 0.032), they were transferred with more embryos when compared with the other patients (2.16, 2.08, and 2.06; *P* = 0.033). However, OPR was still the lowest in patients with thin endometrial thickness (31.58%, 55.56%, and 63.01%;*P* = 0.000) (Table 3). Taken patients with thick endometrial thickness as reference, after adjusting age and number of embryos transferred, adjusted OR for endometrial thickness was 0.284 (95% CI: 0.182–0.444;*P* = 0.000), and 0.734 (95% CI: 0.656–0.822; *P* = 0.000) in patients with thin and medium endometrial thickness, respectively (Table 2).

**Table 3:** Basic characteristics and ongoing pregnancy rate for patients with medium ovarian response.

	Thin	Medium	Thick	*P*
	≤ 7mm	8–13 mm	≥ 14mm	
	Group A	Group B	Group C	
No. of patients	95	4,611	1,795	
Age (y)	31.82 ± 4.78^+^ ^&^	30.64 ± 4.86^+^	30.50 ± 4.91^&^	0.032
BMI (Kg/m^2^)	22.98 ± 3.22	22.49 ± 3.16	22.41 ± 3.00	0.184
Duration of infertility (y)	4.62 ± 3.48	4.53 ± 3.31	4.62 ± 3.33	0.641
No. of oocytes retrieved	9.86 ± 2.47	9.96 ± 2.46	9.87 ± 2.49	0.369
No. of embryos transferred	2.16 ± 0.40^+^ ^&^	2.08 ± 0.28^+^	2.06 ± 0.27^&^	0.033
Endometrial thickness on hCG day	6.47 ± 1.05^+^ ^&^	11.25 ± 1.46^+^ ^Δ^	15.21 ± 1.48^&^ ^Δ^	0.000
Ongoing pregnancy rate (%)	31.58 (30/95)^+^ ^&^	55.56 (2562/4611)^+^ ^Δ^	63.01 (1131/1795)^&^ ^Δ^	0.000

Note: data were mean ± standard deviation unless otherwise noted; BMI = body mass index; hCG = human chorionic gonadotrophin

^+^ Group A versus Group B; *P*<0.05

^&^ Group A versus Group C; *P*<0.05

^Δ^Group B versus Group C; *P*<0.05

For high ovarian responders, the basic characteristics of patients in three groups were comparable in regard to age, infertility duration, and number of oocytes retrieved. Even patients with thin endometrial thickness still tended to have more embryos transferred (2.06, 2.04, and 2.01; *P* = 0.002), the OPR was significantly lower when compared with the other two groups (28.13%, 52.63%, and 63.18; *P* = 0.000) (Table 4). In addition, the association between endometrial thickness and OPR remained significant after adjusting confounding factors (adjusted OR for thin endometrial thickness: 0.233, 95% CI: 0.105–0.514; *P* = 0.000; adjusted OR for medium endometrial thickness: 0.651, 95% CI: 0.536–0.791; *P* = 0.000) (Table 2).

**Table 4:** Basic characteristics and ongoing pregnancy rate for patients with high ovarian response.

	Thin	Medium	Thick	*P*
	≤ 7mm	8–13 mm	≥ 14mm	
	Group A	Group B	Group C	
No. of patients	32	1,503	611	
Age (y)	29.97 ± 4.96^+^ ^&^	29.46 ± 4.24^+^	29.34 ± 4.21^&^	0.649
BMI (Kg/m^2^)	21.40 ± 2.52	22.81 ± 3.25	22.53 ± 2.96	0.012
Duration of infertility (y)	3.13 ± 1.89	4.13 ± 2.99	4.24 ± 2.85	0.127
No. of oocytes retrieved	17.91 ± 4.00	17.84 ± 3.55	17.73 ± 2.85	0.767
No. of embryos transferred	2.06 ± 0.25	2.04 ± 0.22^Δ^	2.01 ± 0.19^Δ^	0.002
Endometrial thickness on hCG day	6.45 ± 0.69^+^ ^&^	11.17 ± 1.44^+^ ^Δ^	15.21 ± 1.45^&^ ^Δ^	0.000
Ongoing pregnancy rate (%)	28.13 (9/32) ^+^ ^&^	52.63 (191/1503) ^+^ ^Δ^	63.18 (386/611)^&^ ^Δ^	0.000

Note: data were mean ± standard deviation unless otherwise noted; BMI = body mass index; hCG = human chorionic gonadotrophin

^+^ Group A versus Group B; *P*<0.05

^&^ Group A versus Group C; *P*<0.05

^Δ^Group B versus Group C; *P*<0.05

## Discussion

To the best of our knowledge, this study is the largest in regards to sample size that explores the effect of endometrial thickness on IVF outcome from one center. Our study demonstrates that a thin endometrial thickness on hCG administration day is associated with significantly lower OPRs in patients with different ovarian response.

Indeed, many studies showed that thin endometrial thickness on the day of hCG administration negatively affects pregnancy rate. At the same time, it is also know to all that, patients' basic characteristics (especially female age, and embryo quality) play very important roles in IVF outcome. In a recent Meta analysis, which included 10,724 patients from 1,170 studies, OPR in patients with endometrial thickness ≤ 7mm was significantly lower compared with that in patients with thicker endometrial thickness. However, the authors also stated that, for those patients with thin endometrial thickness, they had significantly advanced age, and lower number of oocytes retrieved [4]. In addition, embryo quality was not known in most studies, not to mention the fact that endometrial thickness could be measured in several different time points (hCG administration day, embryo transfer day, ect). Thus, we may conclude that, it is other factors such as advanced maternal age, relatively poor embryo quality that should be responsible for the low OPR in thin endometrial thickness patients, but not thin endometrial thickness itself.

Compared with other similar studies and the recent Meta analysis, the current study does have some advantages. Firstly, all patients were from our center, and were treated with standard GnRH-a long protocol. Secondly, endometrial thickness was measured on the day of hCG administration in fresh IVF cycles. In order to control embryo quality, we only include patients transferred with high quality cleavage stage embryo 3 days after oocyte retrieval. More importantly, we compared OPR in the three endometrial thickness groups in poor, medium, and high ovarian responders, respectively, trying to control as many confounding factors as we could.

We firstly drew a general figure to explore the relationship between endometrial thickness and OPR in all the included patients. Fig 1 clearly showed a positively association between these two parameters regardless of ovarian response. Our data not only showed that thin endometrial thickness adversely affects OPR, but also implied that patients should not worry about thick endometrial thickness on the day of hCG administration, because patients with thick endometrial thickness all had significantly higher OPRs in the three groups. Thus, in the following logistic regression analysis, we took thick endometrial thickness (≥14mm) as reference.

For patients with different ovarian response, maternal age, and number of oocytes were comparable in the three endometrial thickness groups. Moreover, patients with thin endometrial thickness had more embryos transferred, but the OPR was the lowest. The adverse effect of thin endometrial thickness on OPR was furthered confirmed by multivariate logistic regression analysis.

The reason why a thinner endometrium results in unsuccessful implantation was still unclear. One possible mechanism was proposed by Casper RF [14]. It was presumed that the oxygen tension near the endometrium surface is lower than that near the basal layer. When an endometrium thickness is <7mm, the functional layer is thin or absent. Implanting embryo would be much closer to the high oxygen tension area, which may have detrimental effect on implantation. However, this was just a conjecture, and should be validated with more studies in the future.

The current large sample size retrospective cohort study has been improved in many ways compared with earlier studies; however, it still has some limitations. First of all, even though all the ultrasound machines are the same, endometrial thickness is measured by different physicians. This may bring some errors in patients with medium or thick endometrial thickness. For a thin endometrium, it is comforting that we measure the endometirum several times in one patient. In addition, even all the patients were transferred with high quality cleavage stage embryos, embryo quality score was not given by the same embryologist.

Taken together, our large sample size study shows that, for patients with different ovarian response, thin endometrial thickness adversely affects OPR. In addition, our data does not show any detrimental effect of thicker endometrium (≥14mm) on IVF outcome.

## Supporting Information

S1 STROBE Checklist (DOC)
